# Preclinical Evaluation of Fisetin in the Management of Diabetic Foot Ulcer in Wistar Rats

**DOI:** 10.2174/0115733998367395250515130758

**Published:** 2025-06-16

**Authors:** Aniket Gupta, Rishabh Chalotra, Randhir Singh

**Affiliations:** 1 Laboratory of Neuroendocrinology and Metabolic Disorders, Department of Pharmacology, Central University of Punjab, Bathinda, India

**Keywords:** Diabetic foot ulcer, fisetin, wound healing, phytochemical therapy, preclinical study, streptozotocin-induced diabetes

## Abstract

**Aim:**

Preclinical Evaluation of Fisetin in the Management of Diabetic Foot Ulcer in Wistar Rats.

**Introduction:**

Diabetic foot ulcer (DFU) is a complication of diabetes mellitus, often leading to non-traumatic amputations and significantly impacting patient morbidity. Globally, the prevalence of DFU ranges from 9.1 to 26.1 million annually. A 2022 meta-analysis revealed that 6.3% of diabetic adults (33 million) are affected by DFUs. The current treatments primarily focus on topical treatments, neglecting the underlying metabolic conditions.

**Objective:**

To investigate the wound healing efficacy of the phytoconstituent fisetin, administered orally, in managing DFU in diabetic neuropathic Wistar rats.

**Methods:**

This study investigates the therapeutic potential of a phytoconstituent fisetin, in the management of wound healing in STZ-NAD induced diabetic animal model with surgically induced wounds after indication of neuropathy. Fisetin was administered orally at doses of 5, 10, and 15 mg/kg for 30 days following the induction of foot ulcers, Various parameters were measured, including blood glucose levels, HbA1c, lipid profile, pro-inflammatory cytokines, antioxidant activity, MDA, and histopathological analysis of wound healing.

**Results and Discussion:**

Fisetin, particularly at 15 mg/kg, significantly modulates blood glucose level, HbA1c, lipid profile, and pro-inflammatory cytokines, further enhancing antioxidant activity, while reducing MDA, indicating a reduction in oxidative stress. Histopathological analysis demonstrated enhanced wound healing by increased fibroblast proliferation and collagen formation, as well as restoration of the epithelial layer. Fisetin also exhibited potential in enhancing re-epithelization, enhancing pro-angiogenic markers, diminishing inflammation, and reducing wound size.

**Conclusion:**

Fisetin demonstrates promising potential in managing DFU by modulating metabolic conditions, reducing blood glucose, oxidative stress, and inflammation, and promoting wound healing. Future studies should focus on unraveling the detailed molecular pathways involved in fisetin's action, along with clinical trials to validate its efficacy in DFU patients.

## 1 INTRODUCTION

Diabetes mellitus is a metabolic disorder characterized by upregulation of blood sugar level, which cause alterations in the physiological system and abnormality in the biochemical processes [1, 2]. In DM, various cellular and metabolic pathways (like the insulin signaling pathway, AMPK pathway, and PPAR) get disrupted, and further, the functional activity of the pancreas to release insulin is decreased [3].

Hyperglycemia often leads to microvascular complications, which affect the blood circulation in vital organs like nerves, kidneys, heart, and lungs [4]. These microvascular complications affect the extremities and disrupt the blood circulation, causing diabetic foot ulcers. DFU often results in malodorous ulcers and can lead to amputations in severe cases [5]. This occurs due to impaired circulation, which leads to a range of pathological conditions, including neurological disorders, peripheral neuropathy, cardiovascular disease, cerebrovascular events, and diabetic retinopathy [6]. There are some risk factors, like food habits, obesity, and ageing in the population, that increases the prevalence of hyperglycemia [7]. Estimated data for 2021 revealed that nearly 537 million people aged between 20 to 79 have been diagnosed with diabetes symptoms, and the number is increasing exponentially every year [8].

The poor management of diabetes further leads to severe complications like neuropathy and foot disorders, like ruptured skin of the lower limb or diabetic foot ulcer. DFU represents a severe stage of diabetic complications primarily caused by advanced glycosylated end products, hyperglycemia-induced neuroinflammation, ischemia, and oxidative stress [9, 10]. These factors contribute to tissue damage in the lower limbs, further aggravating immune dysfunction, poor glycemic control, impaired circulation, and nerve damage. The minor trauma in such tissues can easily progress to ulcers, if untreated, may result in amputations [11]. Neuropathy is a main initiator of DFU development, characterized by sensory impairment that prevents patients from feeling injuries, and often accompanied by peripheral artery disease (PAD), which restricts the blood flow [12]. Neuropathic ulcers are painless and commonly occur on the weight-bearing areas of the foot, while neuro-ischemic ulcers are irregular lesions that occur in patients with both nerve and vascular damage. The risk of both PN and PAD increases with the duration of diabetes [13]. DFU is a leading cause of amputation in diabetes patients, accounting for over 80% of non- traumatic amputations. The mortality and morbidity rates associated with DFU are high. In countries like India, where more than 32 million people live with diabetes, approximately 40,000 amputations occur annually due to DFU complications [14].

The wound-healing process in DFU involves a complex interplay of cytokines, inflammatory mediators, and chemokines, which regulate tissue regeneration and remodeling without directly altering the progression of diabetes. Chronic DFU is characterized by impaired healing, where dysregulation in these factors hampers skin tissue repair [15]. Despite several ongoing pre-clinical and clinical studies, no oral medication has been approved for the specific management of DFU. Present therapies involve cleaning and debridement of the wound, management of infection by suitable anti-infective agents, most preferably topical antiseptics, and off-loading to reduce pressure on the wound [16]. However, so far, these methods do not provide adequate efficacy or cost-effectiveness and offer only symptomatic relief without addressing the underlying causes of the pathological condition. By reviewing the literature, a phytoconstituent fisetin, emerged as a potential candidate for managing hyperglycemia and related complications like diabetic neuropathy. Fisetin (3,3′,4′,7-tetrahydroxyflavone) is a natural flavonoid found in plants like smoke tree (*Cotinus coggygria*), some fruits and vegetables like grapes, strawberries, onions, apples, and cucumbers [17]. It has significant antioxidant and anti-inflammatory effects, which are crucial in the management of DFU. It scavenges ROS, mitigating oxidative stress and preventing tissue damage, which could help in wound healing. Additionally, fisetin also has a reported role in modulating pro-inflammatory cytokines, which are associated with chronic inflammation in DFU [18]. Despite these promising attributes, to date there is no study confirming its role in the treatment of DFU. The present study is designed to investigate the therapeutic potential of fisetin in the management of diabetic foot ulcer in laboratory animals.

## 2 MATERIALS & METHODS

### 2.1 Materials

Streptozotocin and trichloroacetic acid (TCA) were procured from SRL (India), while fisetin was obtained from Sigma-Aldrich (USA). Eosin and hematoxylin were procured from Thermo Fisher Scientific, and 5,5'-dithiobis (2-nitrobenzoic acid) (DTNB) and nicotinamide were also acquired from SRL (India). All chemicals used were of analytical grade to ensure experimental accuracy and reproducibility.

### 2.2 Animal Procurement

A total of 30 adult Wistar rats of either sex, 6 - 8 weeks old, weighing 180 ± 20 g were used in this study, and the study was approved by the Institutional Animal Ethical Committee (IAEC) (CUPB/IAEC/2024/15 on 23/01/2024). The animals were procured from Lala Lajpat Rai Veterinary University of Hisar (Haryana, India) and housed in polypropylene cages with rice husk bedding. Animals were maintained under standard conditions, including a temperature of 25 ± 2 °C, and relative humidity of 60 - 70% with 12 hr. light/dark cycle. The animals were fed a standard pellet diet with regular access to water [19].

### 2.3 Methods

#### 2.3.1 Diabetes Induction

Diabetes was induced in rats by streptozotocin (50 mg/kg *i.p*,), 15 minutes after the administration of nicotinamide (230 mg/kg *i.p.*) [20]. The STZ solution was freshly prepared by dissolving it in ice-cold citrate buffer (pH 4.5) just before use. Before the administration of STZ, rats were fasted overnight with free access to water. To prevent hypoglycemic shock, following STZ administration, the rats were given a 10% glucose solution for 2-3 days. Animals with fasting blood glucose levels of ≥ 250mg/dL after 72hrs. were considered diabetic [21].

#### 2.3.2 Experimental Design and Plan of Work

Wistar rats of similar body weight, age, and gender were selected and randomly distributed based on a simple randomization method. These animals were divided into five groups (n=6). The study followed a single-blinding protocol, where only the corresponding author or Principal Investigator was aware of the group allocations and treatment coding. These groups included a normal control group, a diabetic control group, and three diabetic groups treated with fisetin at 5mg/kg dose, 10mg/kg, and 15mg/kg. Fisetin was suspended in 0.5% carboxymethylcellulose (CMC) in saline. Fisetin was administered orally for 30 days following the induction of foot ulcers. After confirmation of diabetes, the animals were maintained on a normal diet and water during the experimental period. On the 59^th^ day, blood glucose and behavioral parameters were measured to indicate diabetic neuropathy (DN). After the confirmation of DN, the wound was created, and treatment with different doses of fisetin was given for the next 30 days (Fig. **1**).

#### 2.3.3 Behavioral Parameters

Behavioral parameters were observed on the 59^th^ day after induction of diabetes for assessing the neuropathic sensory dysfunction, a key factor in the development of diabetic foot ulcers. Sensory impairment was measured using various tests, including the hot plate test and tactile allodynia.

##### 2.3.3.1 Hot Plate Test

The hot plate test was performed using Eddy’s hot plate analgesiometer on the 59^th^ day after diabetes induction to determine the thermal threshold or paw withdrawal latency with paw licking and jumping used as evaluation parameters. The hot plate was maintained at a temperature of 55 ± 1°C, and a cut-off time of 15 seconds was considered to prevent tissue damage. The pain response was measured in triplicate with a 15-minute interval between each measurement for each individual animal [22].

##### 2.3.3.2 Von Frey Test

The von Frey test was performed on the 59^th^ day after diabetes induction to determine hypersensitivity. The animals were acclimatized for 20-30 min; then the stimuli were given using von Frey filaments (0.4 g - 64 g) to determine the mechanical threshold for paw withdrawal response. The force was applied perpendicularly to the paw surface, either eliciting an immediate paw withdrawal or causing slight bending of the filament held for 1 sec., each stimulation was given five times with a 4-5 sec interval. The cut-off time for each filament was set at 5 seconds, with progressively increasing weight [23].

#### 2.3.4 Wound Creation

Sixty days after STZ administration, rats were anesthetized using ketamine (50 mg/kg) and xylazine (8mg/kg). The skin was disinfected with 70% ethanol, and then a wound was created on the dorsal surface of the foot by removing a section of skin approximately 0.40 cm^2^ using a scalpel. The wound was photographed from day 1^st^ to every 5^th^ day to monitor the healing of the wound.

#### 2.3.5 Biochemical Assay

Various biochemical assays like HbA1c were estimated at the end of the study using the commercially available kit, fasting blood glucose was measured throughout the study using a glucose kit by the glucose oxidase-peroxidase method. The total serum protein was measured by the Lowry protein estimation method. Estimation of lipid profile includes total cholesterol, which was measured using cholesterol oxidase peroxidase biochemical kits, and absorbance was measured at 505 nm. The triglycerides were measured using a glycerophosphate oxidase peroxidase biochemical kit, and absorbance was measured at 546 nm. The HDL was measured using a cholesterol biochemical kit, and absorbance was measured at 505 nm. The very low-density lipoprotein and low-density lipoprotein were calculated using the Friedewald Eqs. (**1**, **2**) [24, 25].

LDL = TC - HDL - TG/5 - (1)

VLDL= TC - HDL - LDL - (2)

#### 2.3.6 Oxidative and Anti-oxidant Parameters

Oxidative stress in the sample was measured by quantifying malonaldehyde (MDA), a marker for thiobarbituric acid reactive species (TBARS) in wound tissue. The absorbance was measured at 532nm and compared with the different standards of MDA concentrations. Whereas the antioxidant level was measured using GSH and SOD. Glutathione (GSH) was estimated through a colorimetric assay involving 5,5-dithiobis 2- nitrobenzoic acid (DTNB), trichloro acetic acid (TCA), and tris buffer. The absorbance was measured at 412nm using a microplate reader. Superoxide dismutase (SOD) enzyme activity was measured using the nitro blue tetrazolium (NBT) method, and the percentage inhibition was calculated to determine the SOD scavenging activity.

#### 2.3.7 Hydroxyproline Assay

Hydroxyproline level was estimated to assess collagen activity in the wound tissue homogenate. The wound tissue samples were autoclaved at 120°C for 20 minutes, followed by adding 450 µL of chloramine-T. The mixture was gently stirred and left stranded at room temperature for 25 min to allow the oxidation reaction to occur. Later, 500 µL of Ehrlich’s reagent (p-dimethyl amino benzaldehyde in n-propanol) was added, and the mixture was incubated at 65°C for 20 min. The absorbance was measured at 560nm using a microplate reader.

Collagen = Hydroxyproline × 7.46

#### 2.3.8 Measurement of Wound Area

The wound area was measured using a wound measurement mobile software, “imitoMeasure - Measure Wounds”, with calibration markers that effectively inspect the wound images and measure the wound's size, area, and chronicity.

#### 2.3.9 Anti-inflammatory Mediators

Cytokines such as IL-6 and TGF-β were measured using rat ELISA kits. The manufacturer's instructions were followed for the assay procedure.

#### 2.3.10 Histological Analysis

The wound tissue specimens were collected and fixed in Bouin’s fixative (15:5:1 of picric acid: formaldehyde: glacial acetic acid) for 24 hours. After fixation, the tissues were embedded in paraffin wax and sectioned into 5µm ribbons, which were mounted on glass slides and stained with hematoxylin and eosin (H&E) for histological analysis of the wound area. The stained tissues were examined under a light microscope at 40x magnification to assess pathological changes in the diabetic foot ulcer samples, comparing the treatment and untreated groups.

#### 2.3.11 Statistical Analysis

The statistical analysis was performed using Graph pad prism software version 8.0.2 and all results were expressed as mean ± standard error of mean. Whereas differences between multiple groups were analyzed through one-way ANOVA and two-way ANOVA, followed by Tukey’s post hoc Multiple Comparison test.

## 3 RESULTS

### 3.1 Behavioral Assessment for DN

On the 59^th^ day, post-diabetes induction, neuropathic sensory dysfunction was evaluated using the hot plate test and tactile allodynia (von Frey test). In the hot plate test, the diabetic control group exhibited increased jumping and licking behavior compared to normal control animals, indicating increased thermal sensitivity. In the von Frey test, the diabetic control animals showed more mechanical hypersensitivity with decreased paw withdrawal latency as compared to the control group. Both the hot plate and von Frey tests indicated the development of neuropathy (Fig. **2**).

### 3.2 Body Weight

The STZ-NAD induced diabetes model shows a significant reduction in body weight as compared to the control group on the 59^th^ day of the diabetes induction. However, with the oral administration of the fisetin, the declining body weight stabilized, and statistical data shows a significant increase in the body weight as compared to the disease group.

### 3.3 Biochemical Estimation

#### 3.3.1 Fasting Blood Glucose

The fasting blood glucose (FBG) level in the diabetic group showed a significant increase. However, after initiation of treatment with fisetin at doses 5, 10, and 15 mg/kg, there was a significant decrease in FBG level compared to the disease control, which was observed on the 75^th^ day and at the end of the study, indicating dose-dependent reduction in FBG (Fig. **3**).

#### 3.3.2 HbA1C

Glycosylated hemoglobin is a critical marker for assessing long-term glucose in diabetes. At the end of the study, the 10 mg and 15 mg/kg fisetin dose groups exhibited a significant decrease in HbA1c level (Fig. **3**).

Estimated average blood glucose (mg/dL) = 28.7 × HbA1c − 46.7

#### 3.3.3 Lipid Profile

The lipid profiles of control, diabetic, and fisetin-treated groups were assessed by determining the total cholesterol, triglyceride, HDL, LDL, and VLDL using biochemical kits in a semi-auto bioanalyzer. After 60 days of diabetic induction, fisetin treatment was administered for the next 30 days, which resulted in a significant reduction in total cholesterol level in all three treatment groups as compared to the control group. Similar results were observed for the serum triglyceride level. Additionally, the HDL was significantly increased in 10mg/kg and 15 mg/kg fisetin-treated groups as compared to the diseased group. Moreover, the levels of LDL and VLDL were significantly decreased in the treatment group as compared to the diseased group (Fig. **4**).

### 3.4 Effect of Fisetin on Inflammatory Mediators

Treatment with fisetin resulted in a significant reduction in IL-6 level in 10mg/kg and 15mg/kg treatment groups as compared to the diseased group. TGF-β plays an important role in regulating angiogenesis by promoting the expression of VEGF (vascular endothelial growth factor). The result shows that the TGF-β level was significantly increased in the diabetic group as compared to the control group, while fisetin treatment at 10mg/kg and 15mg/kg significantly reduced the TGF-β level in a dose-dependent manner (Fig. **5**).

### 3.5 Effect of Fisetin on Oxidative Stress and Antioxidant Activity

MDA level in wound tissue homogenate was elevated in the disease control group compared to the normal control group, whereas treatment with fisetin at doses of 10 mg/kg and 15 mg/kg resulted in a significant reduction in MDA level dose-dependently, indicating decreased oxidative stress. Antioxidant level in wound tissue including GSH and SOD were significantly decreased in disease control group compared to normal control group. However, GSH level was significantly increased at 10 mg/kg and 15 mg/kg, while SOD level similarly increased in 15 mg/kg fisetin treated group (Fig. **6**).

### 3.6 Effect of Treatment on Hydroxyproline Level

Hydroxyproline level was significantly decreased in the disease group as compared to the control group. Fisetin 10 mg/kg and 15 mg/kg treated groups significantly increased the hydroxyproline concentration as compared to the diabetic control group. This concentration of hydroxyproline was expressed as milligrams per gram of tissue sample (Fig. **7**).

### 3.7 Effect of Treatment on Wound Healing

The fisetin-treated groups showed accelerated healing, characterized by the formation of a new epidermal layer and a significant reduction in wound area compared to the diabetic control group. All three doses of fisetin reduced wound size, and the 15 mg/kg dose showed a prominent effect as compared to the diseased group. The images of the wound were captured and analyzed using the imitoMeasure - Measure Wounds mobile software, and wound area measurements were taken every 5^th^ day. A consistent reduction of wound size over time indicated the positive impact of fisetin treatment on the diabetic foot ulcers (Fig. **8**).

### 3.8 Histopathological Examination of Wound Tissue

Histopathological examination of wound tissue was performed after 30 days of fisetin treatment (Fig. **9**). The control group (I) showed regeneration of the epithelial cell layer and fibroblast deposition. Diabetic control group (II) showed no re-epithelization with debris formation, and granulation tissue was observed. The 5mg/kg fisetin treatment group (III) shows marked epithelial regeneration, neovascularization, collagen deposition, and fibroblast proliferation. The 10mg/kg group (IV) and 15mg/kg group (V) rats showed collagen and fibroblast formation along with a reduction in the epithelial layer.

## 4 DISCUSSION

This study investigated the effect of fisetin in treating DFU in laboratory animals. Several studies have highlighted the potential role of fisetin in managing various pharmacological conditions, like a study by Omid-Ali, *et al.* 2024, discussed fisetin as an antioxidant, anti-inflammatory, ability to reduce cell injury, and many others without any major side effects [26]. In the present study, the effect of fisetin on diabetic foot ulcer was investigated. As a member of the fisetin class, fisetin shares the therapeutic potential observed in other flavonoid compounds, which have been reported to play a significant role in the management of DFU [27]. Diabetes was induced by the STZ-NAD model. After the induction of diabetes, there was a decline in the weight of animals and a rise in blood glucose level [28]. STZ leads to β-cell destruction, causing insulin deficiency and hyperglycemia, as a result of which the body’s ability to break glucose molecules is impaired, then the body utilizes fat (lipolysis) and muscle (proteolysis) for energy, resulting in weight loss. Moreover, polyurea causes excessive fluid loss, which can contribute to weight reduction [29]. In this study, treatment with fisetin reversed the weight loss and decreased the elevated blood glucose, which was also confirmed by HbA1c (Glycosylated hemoglobin), a critical marker for assessing long-term glucose in diabetes.

Diabetes, if unmanaged, could develop into diabetic neuropathy. Various studies indicate that 30 to 50 percent of diabetic patients develop diabetic neuropathy in later stages [30]. The STZ-NAD animal model also promotes neuropathy within 5-8 weeks of STZ administration [31]. STZ causes hyperglycemia, which leads to the generation of oxidative stress *via* a redox mechanism [32] and alters various neuropathic pathways [33]. Diabetic neuropathy further contributes to the development of DFUs by loss of protective sensation, impaired healing, and increased risk of infection [34]. In this study, neuropathy was measured after more than 8 weeks or on the 59^th^ day post-STZ administration, using the hot plate and von Frey test. The decreased paw withdrawal latency indicated the neuropathic condition. Foot ulcers were then created using a scalpel on the 60^th^ day, to mimic the pathogenic condition in humans. In this study, treatment with fisetin has reduced the wound size, indicating the role of fisetin in managing DFU. Moreover, fisetin treatment has significantly reduced the oxidative stress which was measured by MDA (important biomarker for oxidative stress) similarly, increased anti-oxidant markers like SOD and GSH. The reduced oxidative stress could facilitate wound healing by preventing damage at the cellular level, improving the functioning of key enzymes and growth factors which are involved in the wound healing process.

STZ-induced diabetic model also alters the lipid profile, promoting atherosclerosis, and impairing blood flow, which delays wound healing. This further increases oxidative stress and inflammation, leading to tissue damage, which ultimately leads to DFU [35, 36]. The treatment with fisetin significantly decreased the total cholesterol, triglyceride, LDL, and VLDL while increasing the HDL level, which helps restore normal blood flow and enhances oxygen and nutrient delivery to the wound site, promoting healing. STZ-induced diabetic foot ulcer also significantly elevates inflammatory markers like IL-6 and TGF-β, primarily due to the combination of physical trauma and diabetic conditions. These inflammatory markers contributed to ulcer progression, impairing immune response, delaying wound healing, and increasing susceptibility to infection [37]. The treatment with fisetin significantly reduced the IL-6 and TGF-β levels. Which helps in managing chronic inflammation, which help balance the immune response, minimizing the tissue damage, and facilitates the proliferation of tissue repair. STZ-induced diabetic model also decreases the hydroxyproline level. Hydroxyproline, a critical component of collagen, plays a vital role in wound healing, and its reduction impairs collagen synthesis, further delaying the wound healing process. Elevated glucose levels disrupt fibroblast activity, reducing collagen production and delaying healing [38]. Treatment with fisetin improved the hydroxyproline level, which improves collagen synthesis, providing structural support to new tissue, which further improves fibroblast activity, restoring collagen production, and accelerating the wound closure process (Fig. **S1**).

These results suggest a potential role of fisetin in treating DFU in modulating various pathological conditions associated with it, further, these results were validated by visual imaging through mobile software (tracks wound size and chronicity), and histopathological examinations (H&E staining and 40x magnification). The outcome of this study demonstrated fisetin's role in promoting the healing of DFUs. Various studies have already reported pharmacological roles of this phytoconstituent, like antioxidant, anti-inflammatory, anti-diabetic, anti-hypertensive, and cardioprotective [39], suggesting its potential for clinical applications in managing diabetic complications and related conditions. Moreover, recent clinical trials also highlighted the use of Vitamin D in the treatment of DFU [40]. Fisetin is a naturally occurring flavonoid and has the potential to modulate oxidative stress, inflammatory responses, and collagen synthesis, which are considered vital parameters in the mechanism of wound healing. While the STZ-induced diabetic rat model is a widely used model to mimic diabetic conditions in pre-clinical experiments, it does not fully replicate the complexity of human diabetic foot ulcers. These necessitate the need for further large-scale studies and clinical trials to confirm the therapeutic potential of fisetin in diverse populations.

## CONCLUSION

This study highlights the potential of fisetin in promoting wound healing in diabetic foot ulcers by mitigating oxidative stress, inflammation, and fibrosis in dose-dependent manner. Fisetin administration at a dose of 15 mg/kg effectively reduced blood glucose, HbA1c, markers of lipid profile, and inflammatory markers like IL-6 and TGF-β. Additionally, it enhanced GSH and SOD level, reduced MDA and promoted collagen formation *via* fibroblast activation. The combined morphological, biochemical, and histological data provided strong evidence for the efficacy of orally administered fisetin in promoting wound healing.

## Figures and Tables

**Fig. (1):**
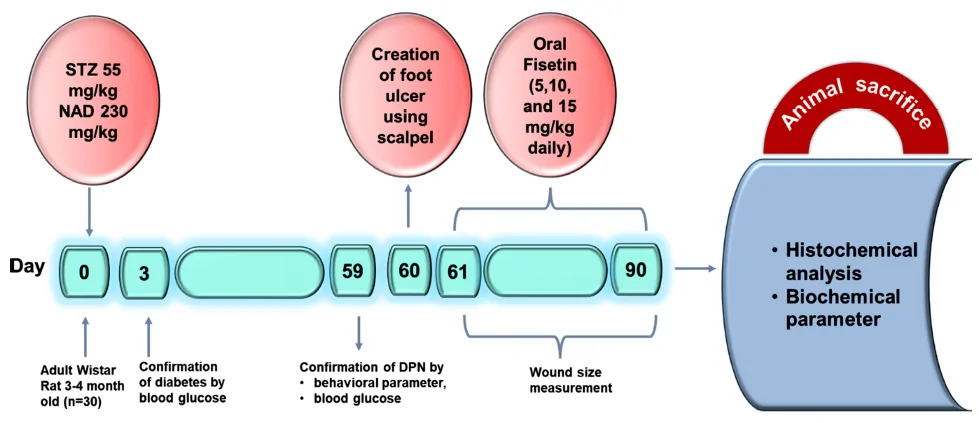
Plan of work.

**Fig. (2):**
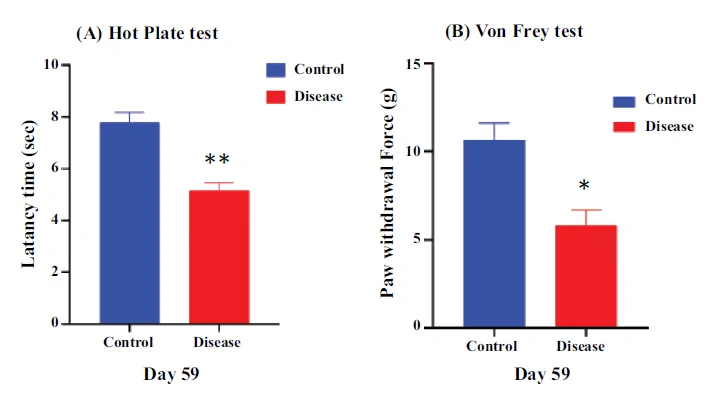
Behavioral assessment (**A**) Hot plate (**B**) von Frey, n = 6. Data represented as * *vs* control and # *vs* disease, *p= 0.0237, **p= 0.0064.

**Fig. (3):**
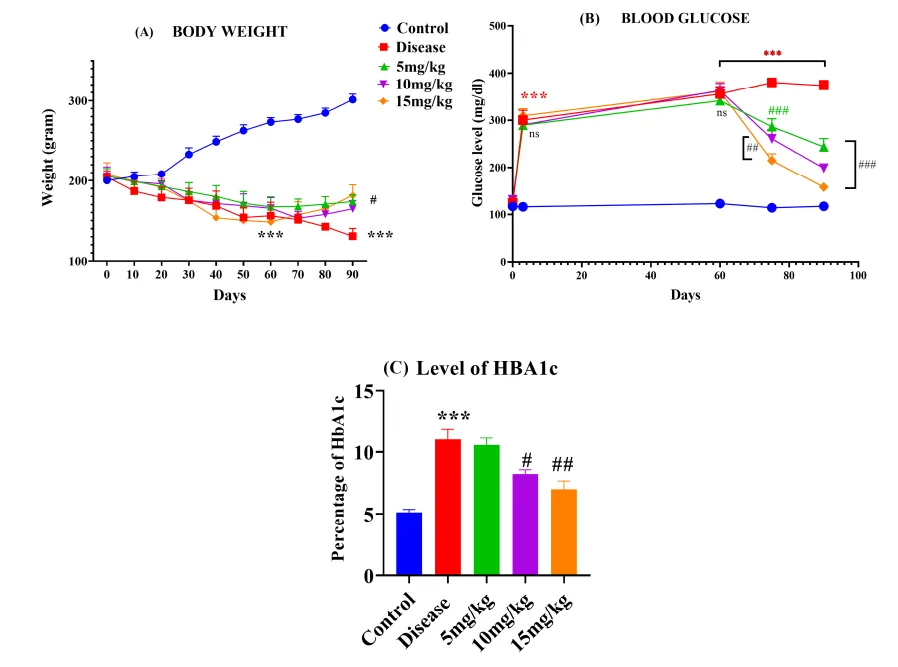
Effect of fisetin treatment on (**A**) Body weight (**B**) Blood glucose (**C**) HbA1c. Data represented as mean ± SEM, n =6. Two-way ANOVA followed by Tukey's post hoc multiple comparison test for body weight and blood glucose, and for HBA1c One way ANOVA Tukey's post hoc test performed, where * *vs* control and # *vs* disease, ***p *<* 0.001, ^#^*p* *<* 0.05, ^##^*p* *<* 0.01, ^###^*p* *<* 0.001.

**Fig. (4):**
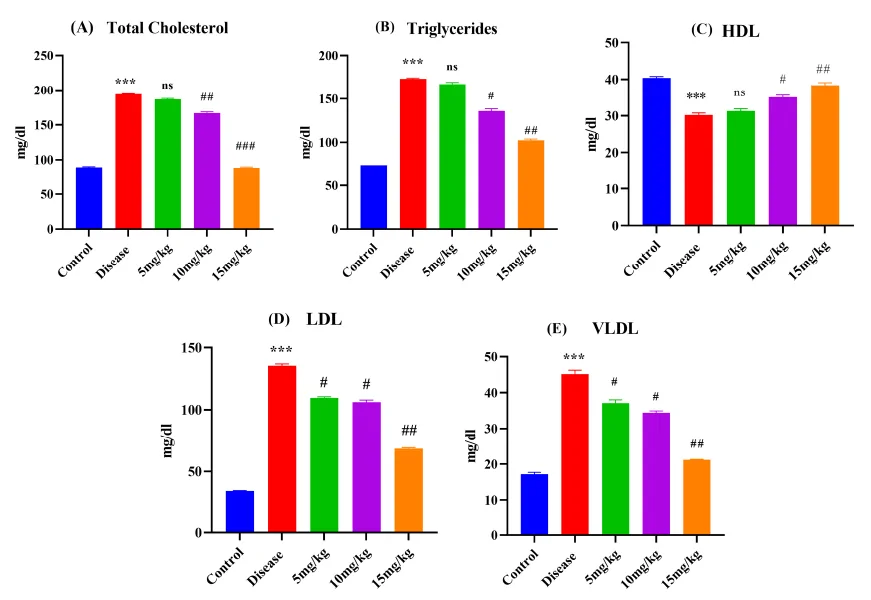
Effect of fisetin on Lipid profile (**A**) Total cholesterol, (**B**) Triglyceride, (**C**) HDL, (**D**) LDL, (E) VLDL. Data represented as mean ± SEM, n=6. One-way ANOVA followed by Tukey's post hoc multiple comparison test. where * *vs* control and # *vs* disease, ***p *<* 0.001, ^#^*p* *<* 0.05, ^##^*p* *<* 0.01, ^###^*p* *<* 0.001, ns = not significant.

**Fig. (5):**
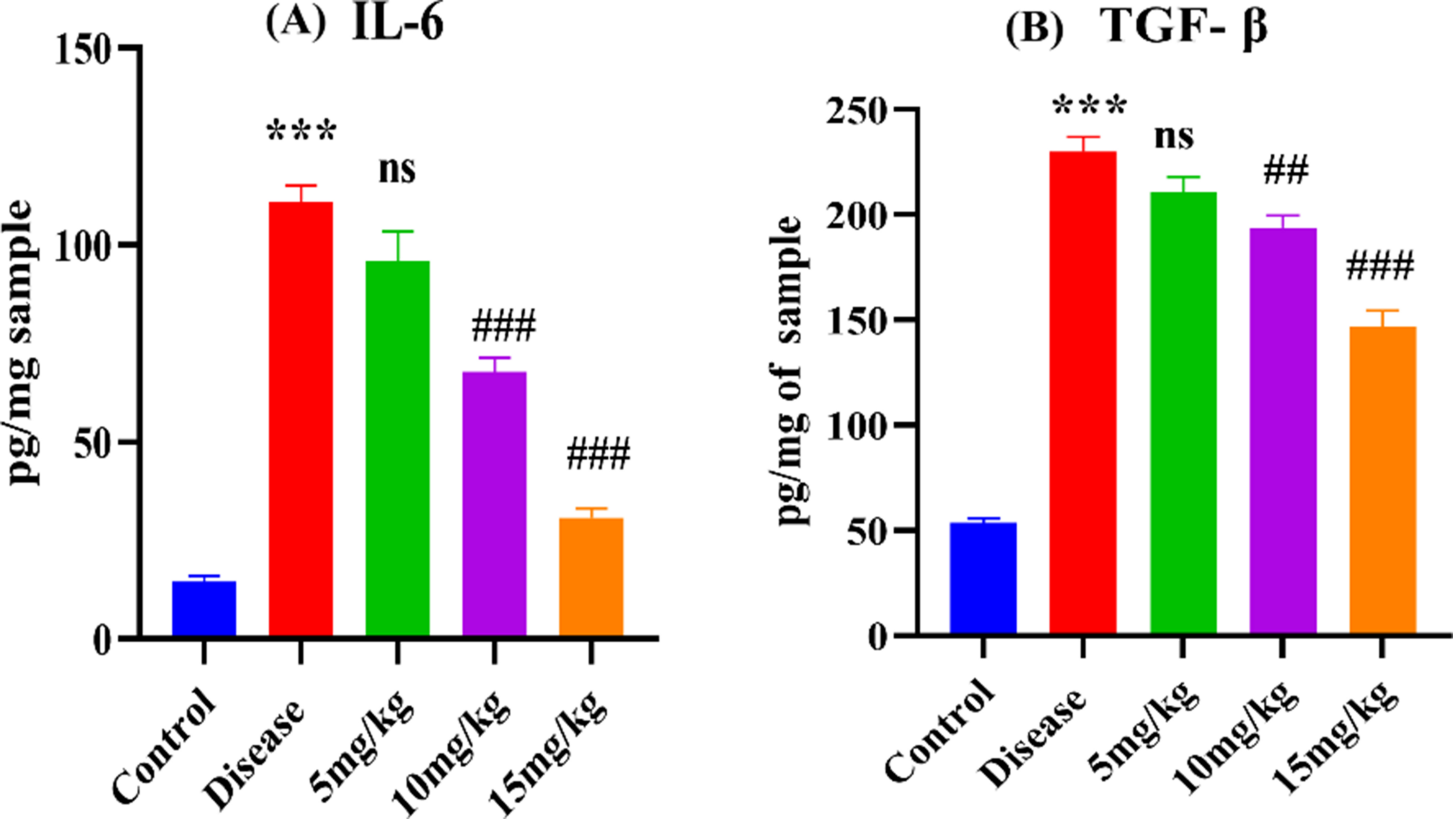
Effect of fisetin on inflammatory markers (**A**) IL-6, (**B**) TGF-β. Data represent mean ± SEM, n =6. One-way ANOVA followed by the Tukey's post hoc multiple comparison test. where * *vs* control and # *vs* disease, ***p *<* 0.001, ^##^*p* *<* 0.01, ^###^*p* *<* 0.001, ns= not significant.

**Fig. (6):**
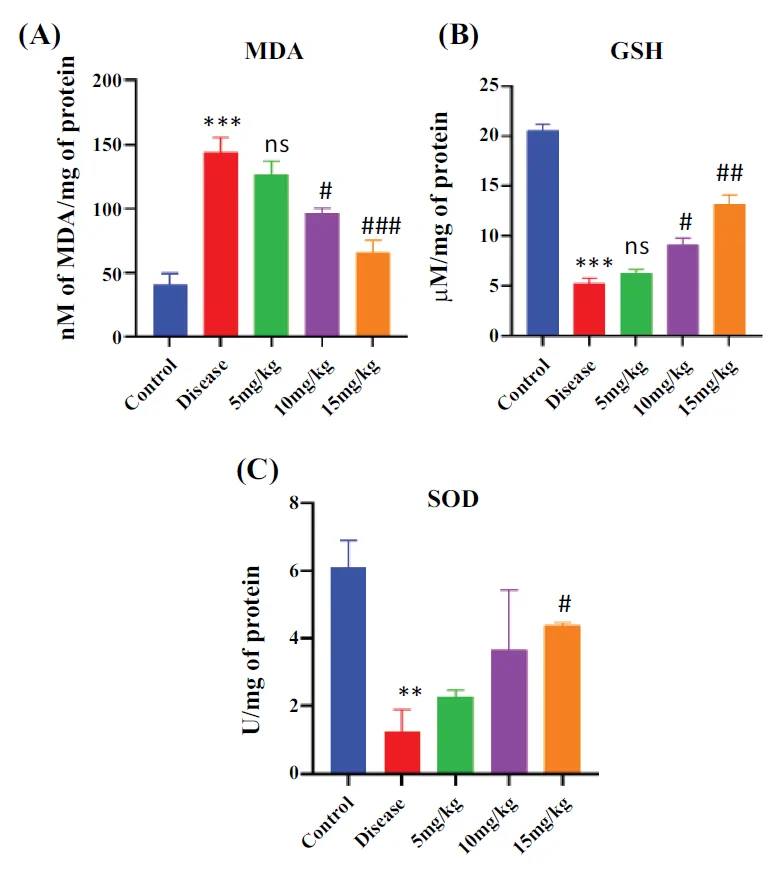
Effect of fisetin on oxidative and antioxidant markers (**A**) MDA, (**B**) GSH, (**C**) SOD. Data represented as mean ± SEM, n =6. One-way ANOVA followed by Tukey's post hoc multiple comparison test. where * *vs* control and # *vs* disease, **p *<* 0.01, ***p *<*0.001, ^#^*p* *<* 0.05, ^##^*p* *<* 0.01, ^###^*p* *<*0.001, ns= not significant.

**Fig. (7):**
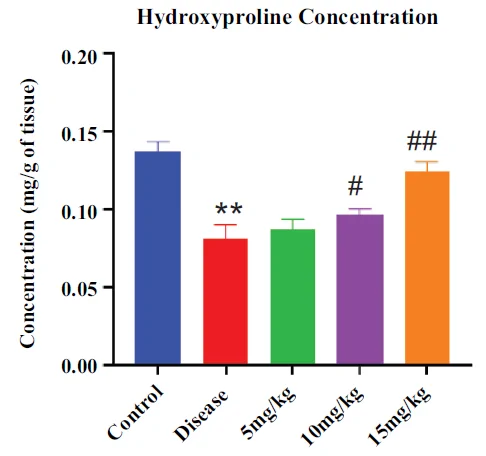
Effect of fisetin treatment on hydroxyproline concentration. Data represented as mean ± SEM, n=6. One-way ANOVA followed by Tukey's post hoc multiple comparison test. where * *vs* control and # *vs* disease, **p *<*0.01, ^##^*p* *<* 0.01.

**Fig. (8):**
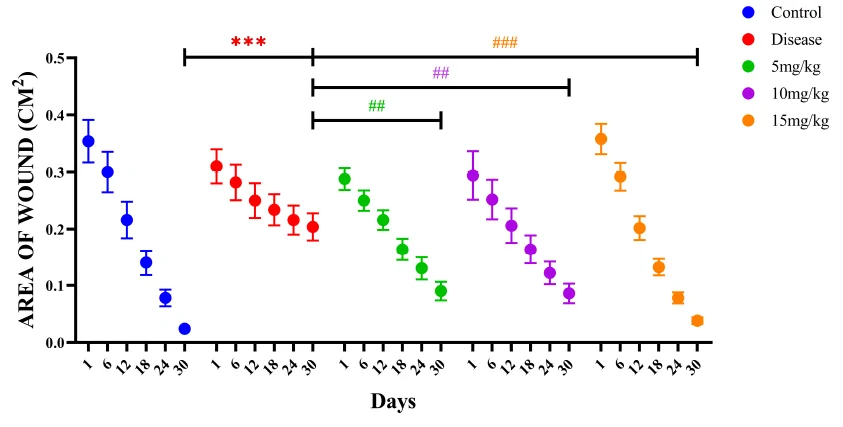
Effect of fisetin on wound area (graphical). Data represent mean ± SEM, n=6. One-way ANOVA followed by Tukey's post hoc multiple comparison test. where * *vs* control and # *vs* disease, ***p *<*0.001, ^##^*p* *<* 0.01, ^###^*p* *<* 0.001, ns= not significant.

**Fig. (9):**
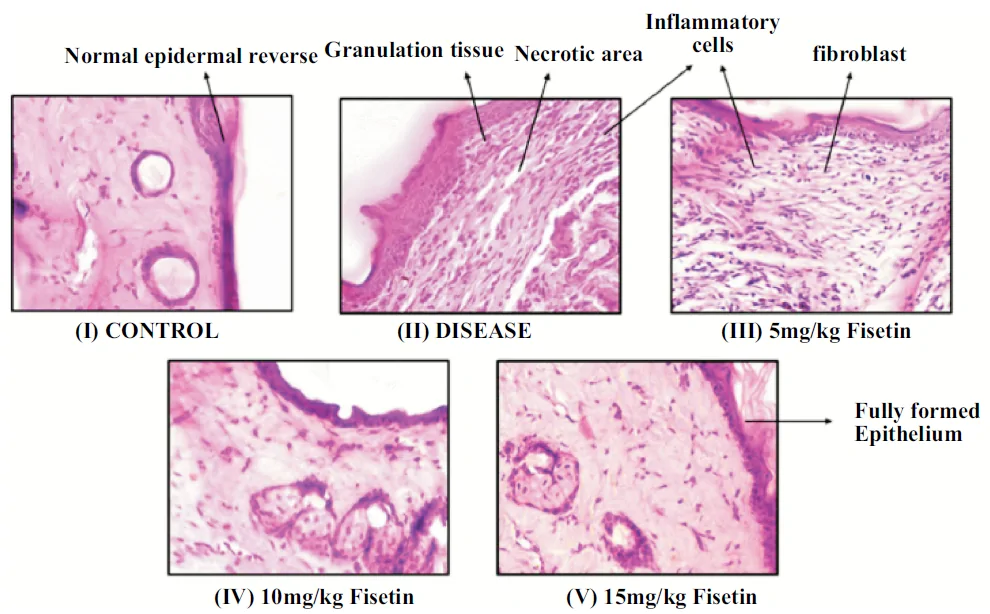
Histopathological analysis using H&E stain on wound sections. The control group shows surface epidermis, while the disease group shows necrotic area and granulations in tissues. In the treatment group, fibroblast formation and reduction in inflammatory cells are evident. The high-dose fisetin group demonstrates a fully developed epidermis. The images were captured and visualized for morphological changes in the wound area at 40X magnification.

## Data Availability

Not applicable.

## LIST OF ABBREVIATIONS

DFU: Diabetic Foot Ulcer
PAD: Peripheral Artery Disease
TCA: Streptozotocin and Trichloroacetic Acid
CMC: Carboxymethylcellulose
DN: Diabetic Neuropathy
FBG: Fasting Blood Glucose
TBARS: Thiobarbituric Acid Reactive Species
MDA: Quantifying Malonaldehyde
