# Supplementary material for: Preclinical Evaluation of Fisetin in the Management of Diabetic Foot Ulcer in Wistar Rats
Source: Curr Diabetes Rev. 2025 Jun 16;22(5):E15733998367395. doi: 10.2174/0115733998367395250515130758 (PMC13714735; doi:10.2174/0115733998367395250515130758)
Supplement: Supplementary file 1 [file CDR-22-5-E15733998367395_SD1.pdf]

Supplementary Material

Preclinical Evaluation of Fisetin in the Management of Diabetic Foot Ulcer in Wistar Rats

Aniket Gupta[1], Rishabh Chalotra[1] and Randhir Singh[1,*]

[1]Laboratory of Neuroendocrinology and Metabolic Disorders, Department of Pharmacology, Central University of Punjab, Bathinda, India

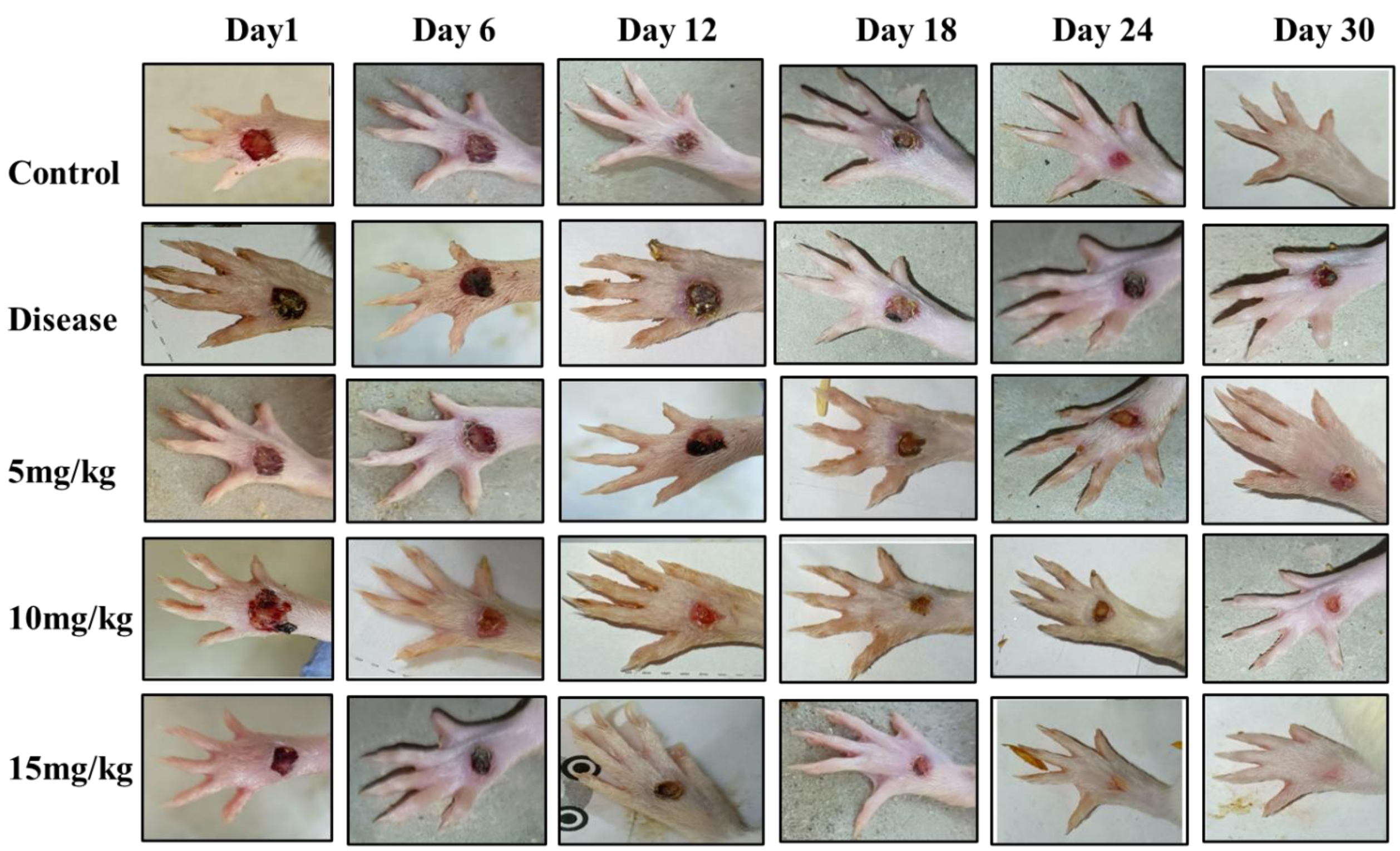

Fig. (S1). Effect of fisetin on wound area.
